# Calculation of the Average Cost per Case of Dengue Fever in Mexico Using a Micro-Costing Approach

**DOI:** 10.1371/journal.pntd.0004897

**Published:** 2016-08-08

**Authors:** Adriana Zubieta-Zavala, Guillermo Salinas-Escudero, Adrian Ramírez-Chávez, Luis García-Valladares, Malaquias López-Cervantes, Juan Guillermo López Yescas, Luis Durán-Arenas

**Affiliations:** 1 Faculty of Medicine, National Autonomous University of Mexico, Mexico City, Mexico; 2 Center for Economic Studies and Social Health, Hospital Infantil de México Federico Gómez, Mexico City, Mexico; 3 Private practice, Mexico City, Mexico; 4 National Commission for Social Protection in Health, Mexico City, Mexico; 5 Faculty of Medicine, National Autonomous University of Mexico, Mexico City, Mexico; 6 Medical Affairs, Sanofi Pasteur Latin America, Mexico City, Mexico; Texas A&M University, UNITED STATES

## Abstract

**Introduction:**

The increasing burden of dengue fever (DF) in the Americas, and the current epidemic in previously unaffected countries, generate major costs for national healthcare systems. There is a need to quantify the average cost per DF case. In Mexico, few data are available on costs, despite DF being endemic in some areas. Extrapolations from studies in other countries may prove unreliable and are complicated by the two main Mexican healthcare systems (the Secretariat of Health [SS] and the Mexican Social Security Institute [IMSS]). The present study aimed to generate specific average DF cost-per-case data for Mexico using a micro-costing approach.

**Methods:**

Expected medical costs associated with an ideal management protocol for DF (denoted ´ideal costs´) were compared with the medical costs of current treatment practice (denoted ´real costs´) in 2012. Real cost data were derived from chart review of DF cases and interviews with patients and key personnel from 64 selected hospitals and ambulatory care units in 16 states for IMSS and SS. In both institutions, ideal and real costs were estimated using the program, actions, activities, tasks, inputs (PAATI) approach, a micro-costing technique developed by us.

**Results:**

Clinical pathways were obtained for 1,168 patients following review of 1,293 charts. Ideal and real costs for SS patients were US$165.72 and US$32.60, respectively, in the outpatient setting, and US$587.77 and US$490.93, respectively, in the hospital setting. For IMSS patients, ideal and real costs were US$337.50 and US$92.03, respectively, in the outpatient setting, and US$2,042.54 and US$1,644.69 in the hospital setting.

**Conclusions:**

The markedly higher ideal versus real costs may indicate deficiencies in the actual care of patients with DF. It may be necessary to derive better estimates with micro-costing techniques and compare the ideal protocol with current practice when calculating these costs, as patients do not always receive optimal care.

## Author Summary

Dengue fever (DF) is caused by infection with the dengue virus, which is spread by the *Aedes aegypti* mosquito. Although the effects of DF are usually mild, in some cases serious illness and even death may result. The average costs per case when extrapolated to society may therefore be high, particularly given the large number of people infected during an endemic year. In Mexico, relatively little is known about the average cost per case (from either the healthcare system or the patient perspective). Such information is important to guide decisions about health policy, e.g. vaccination or public education. We aimed to quantify the average cost per case of DF using a micro-costing approach, both for DF treatment according to an ideal protocol for the management of the patient (´ideal costs´) and according to current treatment practice in the health services (´real costs´). Our results were largely consistent with findings from other international studies, but showed higher ideal costs compared with real costs. We think this may point to inadequate use of laboratory tests and treatments for patients with DF in Mexico. Our cost data will be used in a subsequent publication regarding the economic impact of DF in Mexico.

## Introduction

Dengue fever (DF) is caused by infection with the dengue virus, a single-stranded positive-sense RNA virus of the *Flaviviridae* family [1]. The virus is transmitted almost exclusively by the mosquito vector *Aedes aegypti*, and humans are the only known reservoir for the virus [2, 3]. Clinical presentation varies, with signs and symptoms ranging from uncomplicated fever in the case of simple DF, to bleeding and low platelet counts in the case of dengue hemorrhagic fever (DHF). According to an estimate recently published by the World Health Organization (WHO), between 50 and 100 million infections occur every year [4]. Based on a cartographic approach, Bhatt et al. [2] estimated the number of annual worldwide dengue infections to be 390 million (95% credible interval 284–528 million). The WHO estimated that 500,000 people require hospitalization each year and about 125,000 of those affected die [4].

As in most of Latin America, the disease is widespread in Mexico, although the incidence rate has varied since its reappearance in the 1970s. Peaks in the number of cases occurred in 1980, 1997, and 2009, when more than 130,000 cases were reported nationwide [5]. Between 1995 and 2011, a cumulative total of almost 600,000 cases were reported, with 11% corresponding to DHF. According to the Sub-Directorate General for Epidemiology in Mexico, 62,330, 32,021, and 26,665 cases were reported for the years 2013, 2014, and 2015, respectively [6]. Given these high incidences, the potential seriousness of infections and the considerable disease burden, it is important to have accurate estimates of the average costs associated with the disease. Such estimates will enable efficient allocation of finite healthcare resources [7], for example for vaccination [8, 9], vector control [10–12], and integrated control of dengue through vaccination and vector control [13].

When the present study was initiated, some studies of the economic consequences of dengue had been conducted in Latin American countries including Brazil [14], Colombia [15], and Panama [16]. However, substantial variations in the underlying healthcare context exist in these countries and different methodologies were used in each analysis, such that we cannot extrapolate from these data to other settings. It is thus necessary to standardize and improve the reliability of the methodology and available estimates of the costs of dengue [17, 18].

In Mexico, available data on the costs of the disease are limited. In one of the most comprehensive international studies of the cost of DF, Shepard et al. [19] used specific information from index countries (Venezuela, El Salvador, Guatemala, Panama, Brazil, and Puerto Rico) and estimated the cost of a DF case in countries of the Americas. The cost of an ambulatory case of DF in Mexico was estimated to be US$486 (of which US$264 corresponded to direct medical costs), while the estimated cost of a hospitalized case was US$1,209 (of which US$502 corresponded to direct medical costs). Clearly, the assumptions used when making such an estimate might not hold for Mexico, given the segmentation of the healthcare system and the different organization of each sector within the healthcare system. Secondly, in Mexico there are no cost centers in any of the local healthcare units or in most hospitals. It is therefore not possible to simply aggregate the costs incurred from these types of sources [20, 21]. Thirdly, the two largest public healthcare systems in Mexico are very different in terms of the package of benefits provided, organization of the medical units, as well as the level of resources available and quality of the services provided. The Mexican Social Security Institute (Instituto Mexicano del Seguro Social [IMSS]) provides coverage to all industrial workers and their families. The Secretariat of Health (Secretaría de Salud [SS]) provides coverage to uninsured individuals who do not qualify for IMSS coverage or cannot pay private insurance, and tend to be in a lower income bracket [22, 23]. The proportion of the Mexican population covered by each system is roughly equal (30.4% and 36.6%, respectively) [24].

The co-existence of two different main public healthcare delivery systems (IMSS and SS) is not the only factor that may hamper extrapolation of cost data. In Mexico, the effectiveness of the healthcare sectors has been questioned after multiple reforms have been enacted [25], with patients not always receiving optimal treatment. In particular, the SS healthcare sector, which provides coverage through ‘Popular Insurance’, has suffered from suboptimal funding and fragmented organization (in reality there are 32 different healthcare systems–one for each state in the country) [26]. A recently published study of the economic and disease burden of dengue in Mexico estimated an average cost for DF. The authors used primary data from four major hospitals in the states of Quintana Roo, Morelos, and Tabasco in Mexico [27]. Estimates of DF costs for the two healthcare systems using a larger number of medical units and data from states with endemic DF may be more accurate and will allow comparisons between the two providers.

The main objective of this study was therefore to assess the associated medical costs and cost to the individual with DF using a micro-costing approach to overcome the lack of cost center data. Chart review and interviews with patients and key personnel from 64 medical units in 16 states with endemic DF provided data on costs associated with actual treatment of the disease (‘real costs’). For further comparison we estimated the costs that would be incurred if an ideal treatment protocol were followed (‘ideal costs’).

## Methods

### Ethics Statement

The study was approved by the National Commission of Scientific Research for the IMSS, register number: R-2012 785–070. All participants provided written informed consent and all patient data were anonymized.

### Methodology

The cost per case of DF was calculated by summing the following costs: (1) direct medical costs incurred by healthcare units (including professional services, 127 medical inputs, medical drugs and related products, as well as laboratory tests); (2) costs of dengue from the patient’s perspective (direct medical costs not covered by the public healthcare services and direct nonmedical costs, eg travel expenses); and (3) indirect costs (to the patient and their family) resulting from loss of productivity and loss of earnings. The second and third components include medical and nonmedical direct costs and also indirect costs, such as loss of productivity, caregiver’s costs, etc. All costs were calculated in Mexican pesos and converted to US$ using the exchange rate on September 12, 2012 (1 US$ = 13.03 Mexican pesos) and adjusted for inflation to December 2014 when the analysis was finished.

### Cost Calculation

A hierarchical micro-costing approach was used to calculate direct costs incurred by the healthcare services (known as a program, actions, activities, tasks, inputs [PAATI] analysis) [26, 27]. This type of analysis has previously been used for economic assessments of malaria [28] and hemodialysis in Mexico [26]. The method essentially involves identifying the tasks and inputs from an ideal protocol or current treatment practice for DF and then assigning a unit cost to each. The unit costs were obtained from official IMSS and SS databases. Treatment costs are different for each institution because they have different unit costs for the inputs (see Table 1; full details of the individual unit costs included in the calculation are available in the S1 Appendix).

**Table 1:** Information sources for costings.

Type of cost	Development	Information sources from official databases
**Cost to healthcare services (SS, IMSS, or private sector) using the ideal protocol management**	Task and inputs from ideal protocol validated by expert consensus **+** Unit cost^a^ from official databases (SS, IMSS, or private sector)	• **General purchasing information** • **COMPRANET Website** https://compranet.funcionpublica.gob.mx/web/login.html • **Mexican Social Security Institute (IMSS)** http://compras.imss.gob.mx/?P=search_alt • **Secretariat of Health (SS)**www.csg.salud.gob.mx • **Private sector services**Quotation from private services
**Cost to healthcare services (for SS and IMSS only) using real treatment data from medical units**	Average from task and input actually performed obtained by review of medical histories and from interviews with doctors and hospital administrators, cross-referenced with databases of official prices and costs (see above) **+** Unit cost^a^ from official databases	
**Costs to patients and caregivers**	Interviews with patients	Patient or caregiver

^a^Full details of the individual unit costs included in the calculation are available in the S1 Appendix.

**Fig 1:**
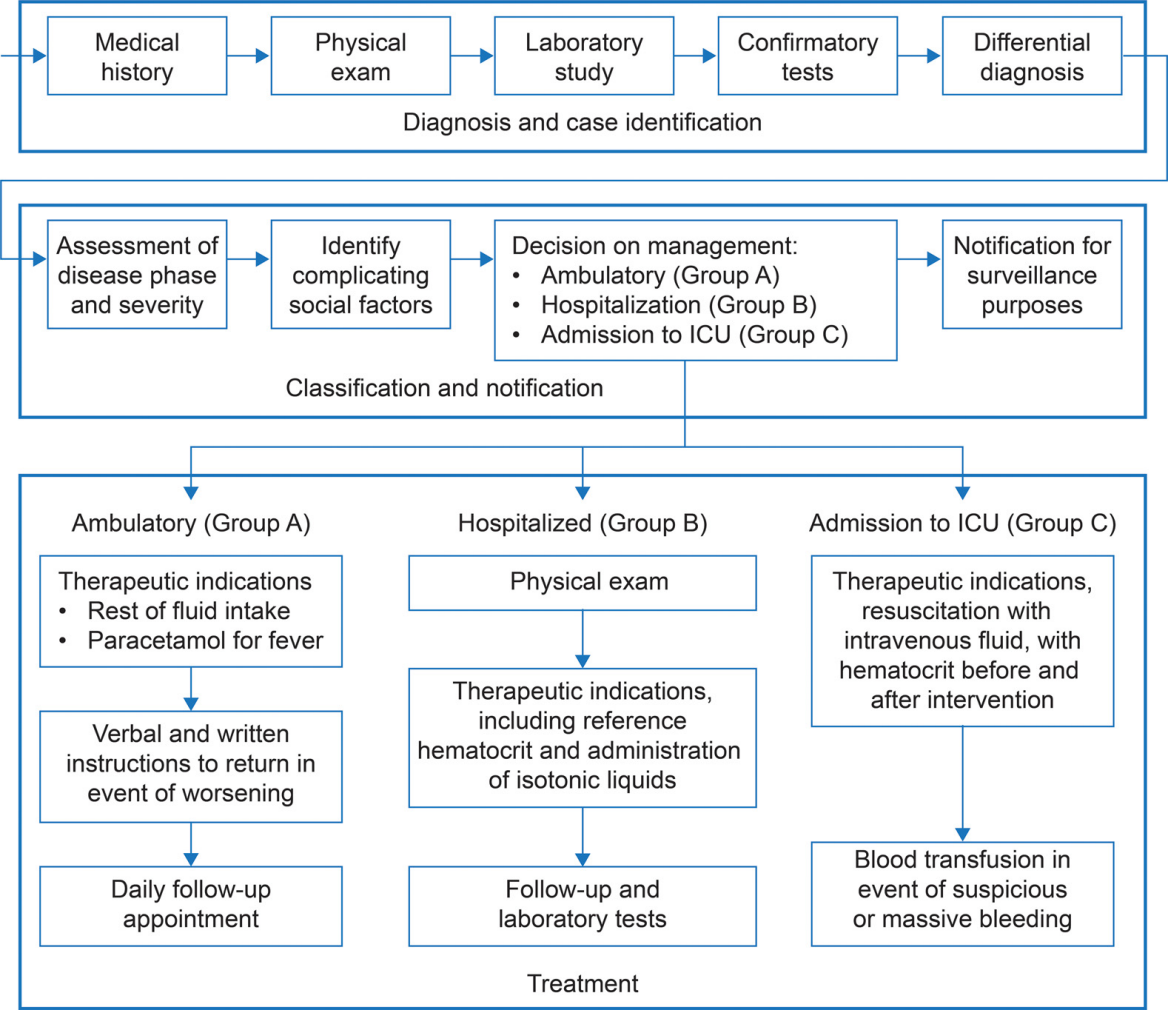
Overview of ideal protocol for treatment of dengue fever and dengue hemorrhagic fever in Mexico. Exam–examination; ICU–intensive care unit.

The tasks and inputs included in the analysis were defined in two ways. In one scenario, an ‘ideal’ cost was calculated according to the tasks and inputs included in an ideal management protocol for patients with DF. Fig 1 outlines the protocol, which was validated in an expert consensus meeting held on June 5, 2012. The protocol was prepared using a systematic review of the literature, as well as guidelines available for Mexico and Latin America. These materials were also used in an expert group discussion of the issue sponsored by the Ministry of Health, in which the authors of this paper actively participated (see Betancourt-Cravioto et al. [29] for further details). Essentially, the clinical protocol is divided into three sections (diagnosis and case identification, classification and notification, and treatment), and each section is assigned a series of activities, which in turn are assigned tasks and inputs.

**Fig 2:**
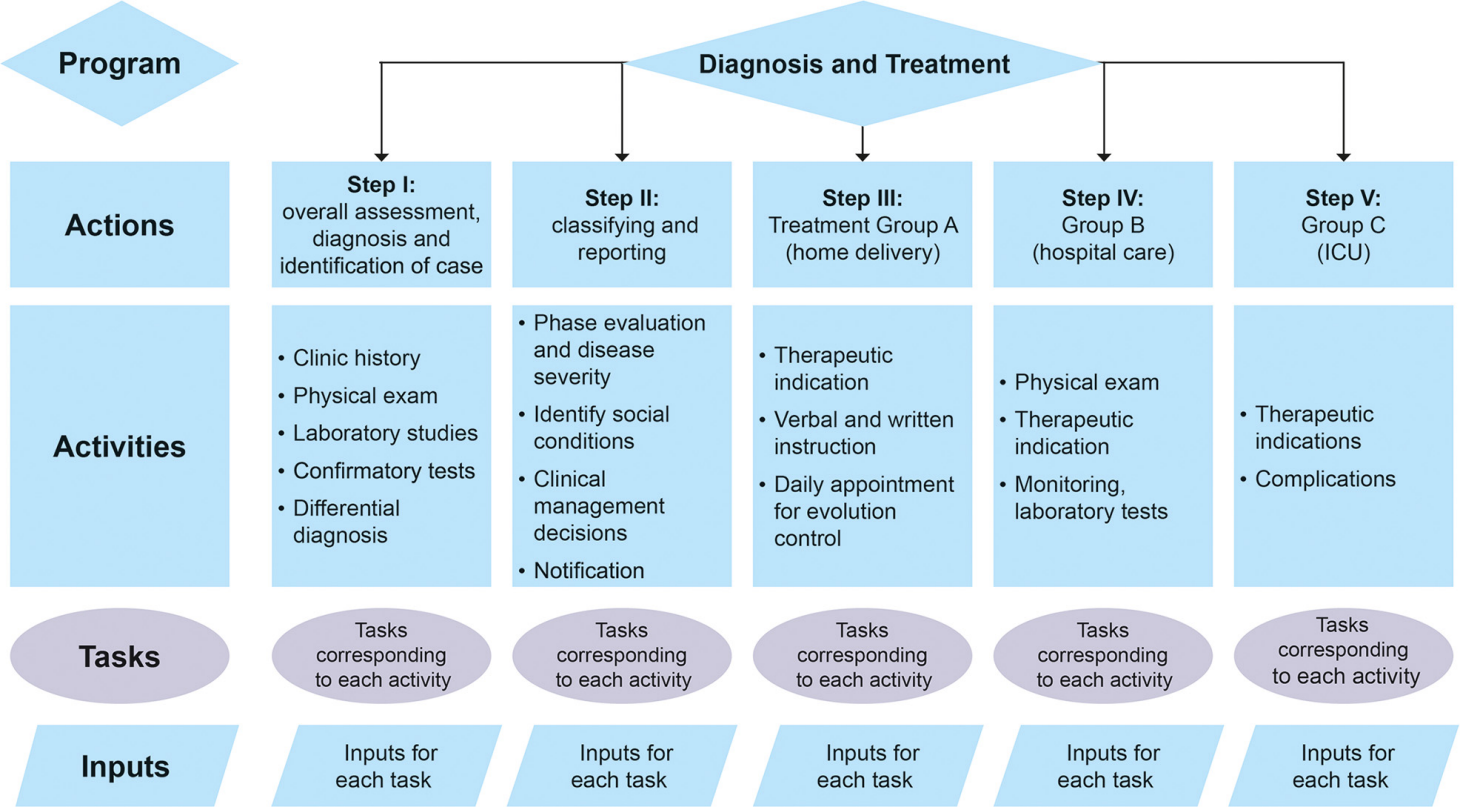
Summary of the PAATI approach. Exam–examination; ICU–intensive care unit; PAATI–program, actions, activities, tasks, inputs.

In the second scenario, treatment costs associated with current practice (which we denote ‘real costs’) in the healthcare services were determined by calculating costs for the tasks and inputs actually performed. These tasks and inputs were determined by chart review and patient interviews. Where data from the medical units were missing or incomplete, the information was supplemented as far as possible from interviews with treating physicians and hospital administrators. The PAATI approach is summarized in Fig 2. The cost of each activity was calculated using the average use reported per input (ie resource or cost type). We looked at each separate type of cost incurred, which allows control of the variability for each component of the healthcare process, rather than using other methodologies where the average overall cost per patient is calculated and then assigned to each activity. For example, using the patient’s clinical chart, the number of blood biometry procedures the patient had undergone was assessed and the average per patient was calculated, which in this case was 1.6 times ± 1.1 per patient. This average was then adjusted by the number of patients in each care setting (outpatient, hospitalized, or intensive care unit [ICU]) who used that resource. The resulting adjusted average resource use was finally multiplied by the unit resource cost. In this way the average cost per case was adjusted for variability by taking into account the proportion of patients who reported consumption of inputs within the sample of patients with DF, in both the patient files reviewed and the patient interviews.

Given the different structures and attributes of the two principal sectors of the healthcare system, costs were calculated for both of them. In addition, unit costs in the private sector were used to estimate the cost of the ideal protocol only, as we had no way of evaluating current treatment practice in the private sector, given the difficulties of selecting a sample from the myriad of private healthcare units in the 16 states with endemic DF. Therefore, in the present study, we only assessed operational costs or variable costs, and no estimates were made of sunk costs or other costs, such as overhead costs (eg utilities, administration, etc.).

The indirect costs from the patient´s perspective were calculated based on information derived from interviews with patients. For these data, the methodology used took into account costs reported by the patients, and these were used to calculate the average cost per case, as well as confidence intervals. A bootstrap analysis was performed to assess variability. Where possible, the patients interviewed were the same patients whose charts were reviewed. Indirect cost was defined as loss of earnings associated with the disease (patient and/or caregiver), with information taken either from direct questions or an estimate based on the number of days of work lost multiplied by the net loss of earnings per day, separately for each institution. For example, in patients who reported being employed, the average length of hospital stay in days was multiplied by the average daily salary. The same approach was used for caregivers. A cost was not assigned if the patient or caregiver did not report any income or they were students. For patients under 16 years of age, only loss of earnings for the caregiver was calculated.

### Sample Selection and Statistical Analysis

We selected a non-probabilistic sample. The study sample was drawn from 32 hospitals and 32 ambulatory care units in 16 Mexican states with endemic DF. Within each state, the hospitals and ambulatory care units with the highest number of cases were selected and up to a maximum of 40 cases per hospital or unit were selected at random for chart review. To avoid selection bias reviewers were instructed to use random numbers to select records. For units with fewer than 40 cases, a census was carried out, and all cases were included in the review. Only patients with an infection occurring in 2012 were considered for chart review.

A sample size sufficient to provide reasonably robust data was calculated using the formula:

$$n=\left(N\sigma^{2}Z^{2}\right)/\left(\left[N-1\right]e^{2}+\sigma^{2}Z^{2}\right)$$

Where *n* = sample size, *N* = total population, *σ* = standard deviation, *e* = acceptable sampling error limit (0.05), and *Z* = 1.96 (for a 95% confidence interval). With these parameters, a sample size of 1,440 subjects was obtained.

As discussed above, costs were calculated for both the SS and IMSS systems, and so the sample size calculation was applied to both systems separately, resulting in double the overall number of patients. This sample would be representative of overall proportions in the SS and IMSS systems at a national level, although it does not take into account state-level stratification.

For indirect costs to the patient, a bootstrap sensitivity analysis was conducted to assess the robustness of the estimation of the average input (based on the method proposed by Efron [30]). Bootstrap analyses are used to estimate the distribution, bias, or variance in a statistical sample or analysis, and to estimate confidence intervals or test hypotheses on parameters of interest when the true distribution of those parameters is unknown. For each type of cost the original sample was sampled to get 5,000 bootstrap resamples to provide estimates and 95% confidence intervals for comparison with the original estimates.

## Results

Data were collected from review of 1,293 charts (90% of the target 1,440 charts according to the sample size calculation). From these charts, it was possible to obtain clinical pathways for 1,168 (81% of the target). Some clinical pathways could not be generated because data were incomplete and could not be supplemented by interviews with treating physicians and hospital administrative personnel. In total, 1,168 patients were interviewed for assessment of out-of-pocket expenses and indirect costs. The same patients were interviewed as those with chart review in 80% of the cases. The remaining patients interviewed had no corresponding chart review.

Overall, 53% of the patients with chart review were women. The mean age of the patient population was 27 years and 34.7% were younger than 18 years old.

### Direct Medical Costs to the Healthcare System

As shown in Table 2, the medical cost differed according to setting (based on outpatients, hospitalized patients, and patients in the ICU), regardless of whether ideal or real costs were considered or which healthcare system (SS or IMSS) was used. Of particular note were the marked differences between the ideal and real costs apparent in both systems. The main factor influencing these differences was the cost of professional services (which accounts for approximately 90% of the differences in the case of outpatients and almost 100% in the case of hospitalized patients and those in the ICU). Professional services also accounted for the largest proportion of the medical cost, while the contribution of expenditure on medicines to the overall medical cost was limited. Nevertheless, of the cost types considered, the cost of medicines showed the largest difference (in terms of percentages rather than absolute costs) between the ideal scenario and the real current treatment practice.

**Table 2:** Ideal and real average costs per case of dengue fever in the Secretariat of Health and Mexican Social Security Institute settings.

Care setting	Secretariat of Health (US$)		Mexican Social Security Institute (US$)	
	Ideal	Real	Ideal	Real
**Outpatients**	**165.72**	**32.60**	**337.50**	**92.03**
Professional services	102.67	27.30	257.40	80.47
Medical consumables	5.26	0.41	4.844	0.56
Drugs and related products	3.63	0.20	3.28	0.25
Laboratory tests	54.15	4.67	71.97	10.74
**Hospitalized patients**^**a**^	**587.77**	**490.93**	**2,042.54**	**1,644.69**
Professional services	538.84	429.70	1,978.51	1,581.74
Medical consumables	6.98	10.56	7.23	6.29
Drugs and related products	23.02	1.61	23.02	4.44
Laboratory tests	18.92	49.04	33.76	52.21
**Patients in ICU**^**a**^	**6,786.19**	**5,361.53**	**23,452.63**	**9,374.54**
Professional services	6,664.00	5,331.20	23,300.07	9,320.03
Medical consumables	38.29	1.36	38.97	1.70
Drugs and related products	46.04	1.39	46.04	1.12
Laboratory tests	37.84	27.56	67.53	51.68
**Patients managed in all three settings**	**7,539.68**	**5,885.06**	**25,832.67**	**11,111.26**

Exchange rate on September 12, 2012: 1 US$ = 13.03 Mexican pesos.

ICU–intensive care unit.

^a^The mean duration of hospitalization was 7 days in both systems.

In both the ideal and real scenarios, the costs to the IMSS were greater (by a factor of 2–4) than the costs to the SS across all patient settings. As for differences between ideal and real costs, the main driver was the cost of professional services. Expenditure on medical consumables and drugs and related products showed very little variation between the two systems, while expenditure on laboratory tests was somewhat higher for the IMSS.

The overall costs of the ideal management of patients in the private sector was higher than the corresponding ideal costs in the IMSS for ambulatory patients (US$487.39 vs US$337.50, respectively) and hospitalized patients (US$4,077.81 vs US$2,042.54), while costs for patients in the ICU were similar (US$23,753.19 vs US$23,452.63).

### Costs for Patients

Expenses from the patient’s perspective are presented in Table 3. As can be seen, here we report direct medical costs incurred by the patient as well as direct costs reported by the healthcare units. These costs increased when the patient received care in the hospital setting. The difference was not so marked between hospitalized patients and those who received care in the ICU, with the exception of patients in the SS system.

**Table 3:** Costs of dengue from the patient’s perspective (direct medical costs + direct nonmedical costs + productivity loss) in patients from the Secretariat of Health and Mexican Social Security Institute settings.

Care setting	Average cost (US$; 95% CI)^a^	
	Secretariat of Health	Mexican Social Security Institute
**Outpatients**		
Medical care^b^	16.29 (9.68–26.40)	39.37 (17.30–62.16)
Other related expenses^c^	N/A	N/A
Lost productivity (patient)	N/A	N/A
Lost productivity (caregiver)	N/A	N/A
**Hospitalized patients**		
Medical care^b^	95.92 (67.61–131.48)	95.65 (48.25–164.64)
Other related expenses^c^	75.98 (59.86–95.50)	86.17 (63.20–132.30)
Lost productivity (patient)	95.35 (67.60–133.91)	122.81 (96.05–164.57)
Lost productivity (caregiver)	72.42 (43.92–121.77)	376.01 (122.36–807.60)
**Patients in ICU**		
Medical care^b^	349.19 (11.12–585.31)	199.53 (–^d^)
Other related expenses^c^	258.31 (136.31–411.22)	90.75 (46.11–149.65)
Lost productivity (patient)	(–^d^)	228.70 (46.04–575.59)
Lost productivity (caregiver)	166.09 (–^d^)	124.57 (–^d^)

Given that the samples of the costs were independent and different sizes, a bootstrap analysis was conducted based on 5,000 resamples to assess the average costs and the 95% confidence intervals (refer to methods section for full description).

CI–confidence interval; ICU–intensive care unit; N/A–not available (not addressed in the questionnaire).

^a^Calculated using bootstrap analysis.

^b^Includes ambulatory and hospital care costs.

^c^Includes transport, meals and lodging.

^d^Insufficient patient data for analysis.

Exchange rate on September 12, 2012: 1 US$ = 13.03 Mexican pesos.

In the outpatient setting, the direct medical care costs are almost double for IMSS patients than for SS patients. It is important to remember that these costs are independent from what was spent by the healthcare unit in the treatment of the patient.

We estimated the loss of productivity based on the number of days reported in the patient interviews for general hospitalization or ICU care. The average cost for a hospitalized patient is therefore not the same as for an ICU patient because, among other factors, the average length of stay is longer for a patient who receives ICU care. Data from the national census of population and national health surveys indicate that, on average, IMSS patients are more affluent than SS patients [22, 23]. The study did not collect data on the average income of patients in the two healthcare systems, although it is important to bear in mind possible differences when interpreting the data.

The questionnaire did not address loss of income for outpatients and these data are indicated as not available in Table 3. We consider this a limitation of the study.

In general, indirect costs appeared to be higher for patients in the IMSS system than for those in the SS system, with the exception of costs for patients in the ICU, which tended to be higher for SS patients (Table 3). The indirect costs for patients with DF corresponding to loss of earnings (not related to medical costs for the patient and/or their caregivers) for patients admitted to the ICU were actually lower compared with hospitalized patients (Table 4).

**Table 4:** Indirect costs of dengue fever according to Secretariat of Health and Mexican Social Security Institute settings.

Care setting	Secretariat of Health (US$)	Mexican Social Security Institute (US$)
**Hospitalized patients**	**165.44**	**523.42**
Patient	93.02	105.99
Caregiver	72.42	417.43
**Patients in ICU**	**153.49**	**291.31**
Patient	–	176.20
Caregiver	153.49	115.11

Exchange rate on September 12, 2012: 1 US$ = 13.03 Mexican pesos.

ICU–intensive care unit.

## Discussion

Despite the fact that a non-probabilistic sampling method was employed in this analysis, the extensive chart review and direct interviewing techniques provided a robust estimation of the average cost of treatment of DF in Mexico. The study also provided information according to type of healthcare system used, thus enabling qualitative comparisons.

In this study we tried to address the limitations of other estimations of average cost per case of DF. The available cost estimates in the literature showed great variability in their methodology, using primary data, macro-costing data, patient questionnaires, administrative data, or most commonly, a combination of data sources including some primary data in a highly restricted population. In our study, the use of micro-costing provides a more detailed understanding of the direct medical costs of dengue, and that is one of its major strengths.

Although we used average costs, we did not want to make the assumption that patients from a single medical unit reflect the whole experience of a country. Therefore, in our study the average costs per case were calculated from data collected from medical audits of 64 healthcare units (1,293 chart reviews) and 1,168 patient interviews in 16 states where DF is endemic.

To compare our results with other studies, here we present the original costs and within brackets the dollars adjusted for inflation using the national consumer price index by country and the official exchange rate, annual average for The World Bank for 2014. For ambulatory cases, the direct real medical cost of 2012 US$32.60 (2014 US$35.93) in the SS was lower than the direct medical costs reported by Sheppard et al. for Brazil 2010 US$49 (2014 US$54.05) [19]), and more recently Colombia 2012 US$67 (2014 US$65.18 [15]), whereas the IMSS direct real medical cost of 2012 US$92.0 (2014 US$101.38) was lower than reported in Venezuela 2010 US$118 (2014 US$130.15) [19]) and Panama 2005 US$332 (2014 US$501.65) [16]). With a micro-costing approach, a multicenter Brazilian study reported a confidence interval of US$31 to US$89 2013, (2014 US$33.81 to 2014 US$97.06) [14], similar to the SS and IMSS ambulatory costs in our study. After we had performed our data collection, Undurraga et al. [27] published an estimation of the ambulatory costs associated with DF in Mexico, where derived costs per episode were calculated by combining patient interviews in four major hospitals in the states of Quintana Roo, Morelos, and Tabasco, macro-costing data from two major public hospitals in Tabasco, MoH health and surveillance data, WHO-CHOICE estimates for Mexico, and previous literature on dengue burden. Indirect costs were obtained based on productivity losses by age, considering both the patient and the patient’s caregivers. These authors reported a cost of 2012 US$65.53 (2014 US$72.22) per outpatient visit and the average cost for ambulatory patients was 2012 $451 (2014 US$497.05) thus lying between the SS and the IMSS costs in our study.

The direct medical costs of hospitalized patients, in relative terms, are higher in our study at 2012 US$490 (2014 US$ 539.99) and 2012 US$1,644 (2014 US$1811.71) for the SS and IMSS, respectively, compared with the estimates for Brazil 2013 US$318 (2014 US$346.80) [19]), Colombia 2012 US$330.6 (2014 US$321.63) [15]) and in the new multicenter study for Brazil 2013 US$238–479 (2014 US$259.55–522.38) [14]). Costs reported for Venezuela 2010 US$864 (2014 US$952.99) [19]) and Panama 2005 US$1065 (2014 US$1609.22) [16]) are higher than the SS costs but lower than the cost to the IMSS.

Although comparisons with other countries may be illustrative, firm conclusions cannot be drawn given the differences in economic development, population size, and healthcare systems, as well as the methodology used for the estimates.

In the case of hospitalized patients, comparisons are more difficult because no distinction is made between hospitalized and ICU settings in most Latin American studies. Interestingly, the ideal costs estimated in our study, 2012 US$587 (2014 US$646.88) for SS and US$2,042 (2014 US$2,250.31) for IMSS were closer to the extrapolated costs reported by Shepard et al. [19] for total hospitalized cost, 2010 US$1,209 (2014 US$1,333.53) in Mexico. Undurraga et al. [27] reported that the hospital cost for Mexico was 2012 US$240.04 (2014 US$261.48) per bed day and "the average cost per non-fatal dengue episode was $1,327 for hospitalized patients (2014 US$1,445.53) (direct medical: $1,010 (2014 US$1,100.22); direct non-medical: $174 (2014 US$189.54); indirect: $143 (2014 US$155.77) and $451 for ambulatory patients (2014 US$491.29) (direct medical: $253 (2014 US$275.60); direct non-medical: $92 (2014 US$100.22); indirect: $106 (2014 US$115.47) ". Thus, these data are between the SS and the IMSS costs in our study.

Finally, we are not interested in presenting the virtues or deficiencies of the Undurraga approach; we simply want to clarify the differences with our approach.

Indeed, a particularly striking feature of our results are the differences between the direct medical costs of actual treatment in clinical practice and those that would be generated if an ideal protocol, validated by experts in the field, were followed. The difference was particularly marked for outpatients. The first implication is that cost studies based on an ideal treatment protocol may not reflect clinical reality, at least in Mexico. The difference in personnel costs may reflect systematic differences in either the productivity of the personnel, their training, and treatment standards among the medical units of the institutions included in the study. The second implication is that there may be shortcomings in the Mexican healthcare systems, particularly in the outpatient setting, despite extensive reform in recent years with a view to improving the quality of care [20, 21, 31]. The main driver of the difference between real and ideal costs is the cost of professional services and the use of laboratory tests (including confirmatory tests). This may indicate that the treating physicians are not dedicating sufficient time to their patients, nor providing optimal laboratory follow-up for patients. Furthermore, we note that most of the data were collected in 2012, which was not an epidemic year. In epidemic years, it is likely that overburdening of the system would further accentuate the differences between real and ideal costs, as physicians would be forced to dedicate less time to their patients, and the demand for laboratory tests would be higher.

Another noteworthy feature of the results presented here is the higher direct medical costs incurred within the IMSS system compared with the SS system. Another Mexican study utilizing chart reviews and direct patient interviews estimated the direct medical costs in the IMSS system for patients with osteoporosis and hip fracture [24]. Compared with the SS system, they also found considerably higher cost for IMSS (US$3,891.20 vs US$1,590.70, respectively). The main driver of this difference in the present study was personnel costs, which may reflect differences in pay between the two systems. The IMSS was set up over 60 years ago and has strong unions, which may be responsible for higher staff costs. In addition, there may be a tendency to use the private sector as a guide when setting prices, particularly in the ICU setting, where the IMSS and private sector prices are very similar. As noted by Clark et al. [24] in their study of hip fracture, the SS system, which provides medical care to the lowest income groups, receives larger subsidies that are not otherwise reflected in the costs generated by a micro-costing analysis such as the present one. It is important to note that the results in our study are closer to the multicenter study in Brazil [14] that used a micro-costing approach, even though we have clear differences between our healthcare systems.

The greater availability of treatments in the IMSS system may explain the slightly higher treatment costs in the outpatient and hospitalized settings, although the more fragmented nature of the SS system may increase procurement costs. There is evidence that the heterogeneity of the state healthcare services (SS system) may lead to small variations between areas, resulting in care below that recommended in clinical guidelines. This substandard care is associated with lower costs.

Of note were the expenses reported by the patient beyond those incurred by the healthcare institutions for hospitalized patients and patients in the ICU. Expenses incurred by patients were higher within the SS system, suggesting that these patients had to supplement the care provided by the SS to a greater extent than IMSS patients. Any comparison of the costs in the IMSS and SS systems should bear in mind that the cost of each activity was calculated using the average use reported per input and the unit cost reported for every organization and state. In general, the unit costs in the IMSS system are more stable and better registered at the central level of the organization than costs within the SS system.

A limitation of our study is that loss of earnings due to days off work was only registered for patients admitted to hospital or ICU and thus was not captured for outpatients.

Indirect costs were greater for patients who attended through the IMSS, possibly reflecting their higher socioeconomic status. Interestingly, the difference was greater for hospitalized patients than those admitted to the ICU. This observation can be explained by the fact that family members may be allowed to stay with patients in a hospital ward but not with patients in an ICU setting and they will thus incur more expenses.

A second limitation was that we do not include data on management, electricity, and other utilities in the cost estimation. However, it was considered that the relevant cost is variable cost, and given the lack of data on management, electricity and other utilities, we considered that it would be misleading and add more uncertainty to the cost estimation.

As noted above, a principal weakness of the study is possible bias resulting from the non-probabilistic sampling method employed in this study. The states where DF is endemic were chosen, and, within those states, the centers with the highest number of cases were selected. Furthermore, patients requiring hospitalization and those with DHF would be more likely to attend larger reference centers, so this approach may lead to an over-representation of patients with more severe disease. A further bias may have been introduced by not ensuring that all patients interviewed corresponded to those whose charts were reviewed. However, given that 80% of the patients interviewed also had a chart review, differences would have limited impact on the final average results.

Nonetheless, by concentrating on larger centers with a larger number of cases, it was possible to collect extensive primary data that would otherwise be hard to acquire from probabilistic samples. In essence, statistical robustness was sacrificed to enable the data collected to reflect more accurately clinical practice in the sample, in contrast to many studies of costs of DF in the Latin American region. Additionally, the data available from chart review were not complete (and may not have been entirely reliable) and had to be complemented with other data sources. While this may also compromise the integrity of the data, we believe that chart review, despite its inherent limitations, ultimately provides the best approximation to what actually happens in clinical practice. It is also possible that recall bias was present in the interviews, but for most patients, DF is an event that they are unlikely to forget.

In conclusion, this study pointed to real costs associated with DF in line with those reported in other Latin American countries (with the caveat that direct comparisons should be treated with caution given the differences between countries in economic development, healthcare systems, size, and the cost methodology employed in studies). Of particular note were the large differences in the real costs derived from patient records and the ideal cost calculated from an ideal treatment protocol. This difference perhaps points to deficiencies in the care of patients with DF in Mexico. It also indicates the importance of collecting primary data when calculating DF costs to guide health policy decisions in this and other diseases that lack an appropriate estimate of its cost to the system, which poses a major hurdle for healthcare planning.

## Supporting Information

S1 Appendix Medical costs of dengue fever in Mexico. (PDF)
