# Correction: Calculation of the Average Cost per Case of Dengue Fever in Mexico Using a Micro-Costing Approach

**DOI:** 10.1371/journal.pntd.0005119

**Published:** 2016-11-01

**Authors:** Adriana Zubieta-Zavala, Guillermo Salinas-Escudero, Adrian Ramírez-Chávez, Luis García-Valladares, Malaquias López-Cervantes, Juan Guillermo López Yescas, Luis Durán-Arenas

There are errors in the Funding section. The correct funding information is as follows:

The study was funded by Sanofi Pasteur. The funder established a committee that reviewed every step of the research process, received and approved the protocol, and approved data collection. Editorial assistance with the preparation of the manuscript was provided by a professional medical writer, Greg Morley of MedSense, funded by Sanofi Pasteur.
