# Supplementary material for: Calculation of the Average Cost per Case of Dengue Fever in Mexico Using a Micro-Costing Approach
Source: PLoS Negl Trop Dis. 2016 Aug 8;10(8):e0004897. doi: 10.1371/journal.pntd.0004897 (PMC4976855; doi:10.1371/journal.pntd.0004897)
Supplement: S1 Appendix — (PDF) [file pntd.0004897.s001.pdf]

2.9 Real Medical Costs IMSS

Actions	Description of Input	Unit Measure	Unit cost	Quantity	Partial cost	Subtotal	Total Action 3	Remarks
1 and 2							$1,198.59	
3. Treatment Group Outpatients	**Visit to family doctor (up to 5 visits)**							
	**a) Professional services**					$1,048.60		
	Primary care visit	Session	$559.00	1.600000	$894.40			
	Emergency room visit	Session	$514.00	0.300000	$154.20			
	**b) Medical consumables**					$7.32		
	Multiple sample needle 20 GX1”	Item	$0.26	1.330000	$0.35			
	Antiseptics and germicides, denatured alcohol	Milliliter	$0.03	1.330000	$0.05			
	Disposable gloves	Item	$1.19	2.110000	$2.50			
	Syringes	Item	$0.90	1.330000	$1.20			
	Face masks	Item	$0.13	1.270000	$0.17			
	Sterile gauze	Item	$0.28	4.000000	$1.10			
	Swabs	Gram	$0.06	1.330000	$0.07			
	Blood collection tube with EDTA K2 powder (anticoagulant) LILA	Item	$1.42	1.330000	$1.89			
	**c) Drugs and related substances**					$3.29		
	Paracetamol	Item	$0.20	10.834993	$2.16			
	Amoxicillin	Tablet	$0.48	0.138158	$0.07			
	Amikacin	Vial	$2.19	0.046053	$0.10			
	Benzylpenicillin	Vial	$2.32	0.012336	$0.03			
	Diphenidol	Tablet	$0.13	0.098684	$0.01			
	Benzonatate	Blister	$0.59	0.108553	$0.06			
	Hyoscine butylbromide	Tablet	$0.55	0.108553	$0.06			
	Ciprofloxacin	Tablet	$0.46	0.046053	$0.02			
	Chlorphenamine	Tablet	$0.16	3.013158	$0.48			
	Vitamin B complex	Tablet	$0.11	0.296053	$0.03			
	Diclofenac	Capsule	$0.21	0.046053	$0.01			
	Oral electrolytes	Item	$2.19	0.016513	$0.04			
	Phenazopyridine	Tablet	$0.52	0.049342	$0.03			
	Hydroxocobalamin	Tablet	$0.11	0.006579	$0.00			
	Metamizole	Item	$0.58	0.238487	$0.14			
	Naproxen	Tablet	$0.20	0.059211	$0.01			
	Omeprazole	Tablet	$0.25	0.026316	$0.01			
	Ranitidine	Tablet	$0.16	0.131579	$0.02			
	Ferrous sulfate	Tablet	$0.14	0.065789	$0.01			
	Trimethoprim	Tablet	$0.24	0.032895	$0.01			

...continued

Actions	Description of Input	Unit Measure	Unit cost	Quantity	Partial cost	Subtotal	Total Action 3	Remarks
3. Treatment Group Outpatients	**d) Laboratory studies**					$139.38		
	Hematic biometry	Study	$88.00	1.243421	$109.42			
	Gasometry	Study	$113.00	0.016447	$1.86			
	LFT	Study	$61.00	0.085526	$5.22			
	ES	Study	$61.00	0.036184	$2.21			
	Urinalysis	Study	$61.00	0.085526	$5.22			
	QS	Study	$61.00	0.151316	$9.23			
	X ray	Study	$155.00	0.003289	$0.51			
	Ultrasound	Study	$155.00	0.016447	$2.55			
	Electrocardiogram	Study	$482.00	0.006579	$3.17			
4. Treatment Hospitalization							$21,430.35	
	**Hospitalization**							
	**a) Professional services**					$20,610.08		
	Hospitalization	Days	$5,156.00	3.997300	$20,610.08			
	**b) Medical consumables**					$82.01		
	Tongue blade	Item	$0.06	1.500000	$0.09			
	Blood collection tube with EDTA K2 powder (anticoagulant) LILA	Item	$1.42	1.670000	$2.37			
	Multiple sample needle 20 GX1”	Item	$0.26	1.360000	$0.35			
	Swabs	Gram	$0.06	2.920000	$0.16			
	Antiseptics and germicides, denatured alcohol	Milliliter	$0.03	129.300000	$4.40			
	Disposable gloves	Item	$1.19	2.330000	$2.76			
	Insulin syringe	Item	$0.76	1.570000	$1.19			
	10 ml syringes	Item	$0.90	1.570000	$1.41			
	Surgical soap	Milliliter	$0.23	82.500000	$18.98			
	Sterile gauze	Item	$0.28	3.560000	$0.98			
	Uro-Labstix strip	Item	$1.05	1.750000	$1.84			
	Recipient for urine samples	Item	$1.00	1.000000	$1.00			
	Face masks	Item	$0.13	1.540000	$0.20			
	Foley probe	Item	$16.18	1.000000	$16.18			
	Cistoflo urine bag	Item	$7.38	1.000000	$7.38			
	Lubricating gel	Gram	$0.04	75.000000	$3.00			
	Disposable tissues	Item	$2.71	2.500000	$6.78			
	ROJO Serology tube without anticoagulant	Item	$10.79	1.200000	$12.95			

...continued

Actions	Description of Input	Unit Measure	Unit cost	Quantity	Partial cost	Subtotal	Total Action 3	Remarks
4. Treatment Hospitalization	**c) Drugs and related substances**					$57.91		
	Paracetamol	Item	$0.20	5.057400	$1.01			
	Ambrosol	Milliliter	$0.05	0.056300	$0.00			
	Amoxicillin	Tablet	$0.48	0.032200	$0.02			
	Amplification	Tablet	$0.38	0.032200	$0.01			
	Hyoscine butylbromide	Vial	$2.67	0.020100	$0.05			
	Ciprofloxacin	Tablet	$0.46	0.040200	$0.02			
	Diphenhydramine	Vial	$12.75	0.042200	$0.54			
	Ranitidine	Tablet	$0.16	0.168900	$0.03			
	**Saline solution**<br>**d) Laboratory studies**	Bag	$7.20	7.810000	$56.23	$680.36		
	LFT (Hosp)	Study	$77.00	1.045600	$80.51			
	Qs (Hosp)	Study	$77.00	1.351200	$104.04			
	Gasometry (Hosp)	Study	$113.00	0.324400	$36.66			
	Es (Hosp)	Study	$77.00	0.962500	$74.11			
	X ray (Hosp)	Study	$266.00	0.128700	$34.23			
	Electroencephalogram	Study	$482.00	0.010700	$5.16			
	Tomography	Study	$1,290.00	0.002700	$3.48			
	Echocardiogram	Study	$482.00	0.010700	$5.16			
	Electrocardiogram	Study	$482.00	0.053600	$25.84			
	Ultrasound (Hosp)	Study	$155.00	0.337800	$52.36			
	Hematic biometry	Study	$88.00	2.941000	$258.81			
5. Treatment in Intensive Care Unit							$122,150.27	
	**Intensive care**							
	**a) Professional services**					$121,440.00		
	Hospitalization in ICU	Days	$30,360.00	4.000000	$121,440.00			
	**b) Medical consumables**					$22.23		
	Tongue blade	Item	$0.06	2.500000	$0.14			
	Multiple sample needle 20 GX1”	Item	$0.26	1.000000	$0.26			
	Swabs	Gram	$0.06	3.670000	$0.20			
	Antiseptics and germicides, denatured alcohol	Milliliter	$0.03	204.000000	$6.93			
	Disposable gloves	Item	$1.19	3.800000	$4.50			
	Disposable infusion kit	Item	$2.70	1.170000	$3.16			
	Punzocat 22 g	Item	$5.47	1.200000	$6.56			
	Face masks	Item	$0.13	3.500000	$0.46			
	Adhesive tape	Centimeter	$0.00	1.500000	$0.00			

Actions	Description of Input	Unit Measure	Unit cost	Quantity	Partial cost	Subtotal	Total Action 3	Remarks
5. Treatment in Intensive Care Unit	**c) Drugs and related substances**					$14.64		
	Paracetamol	Item	$0.20	1.200000	$0.24			
	Solutions	Bag	$7.20	$2.00	$14.40			
	**d) Laboratory studies**					$673.40		
	LFT (Hosp)	Study	$77.00	1.200000	$92.40			
	Qs (Hosp)	Study	$77.00	2.000000	$154.00			
	Gasometry (Hosp)	Study	$113.00	0.800000	$90.40			
	Es (Hosp)	Study	$77.00	1.400000	$107.80			
	Hematic biometry	Study	$88.00	2.600000	$228.80			
						TOTAL IMSS	144,779.21	

Input	Source	Unit measure	Presentation	Quantity per presentation	Overall cost	Unit cost	Type
Insulin syringe	IMSS	Item	Item	1.00		$0.61	Consumable
Saline solution	IMSS	Bag	Bag	500.00	$7.20	$7.20	Consumable
Uro-Labstix strip	IMSS	Item	Bottle	100.00	$95.00	$1.05	Consumable
Adhesive tape	SS	Centimeter	0	-	$-	$0.00	Consumable
0.9% saline solution	SS	Milliliter	0	-	$-	$0.02	Consumable
Antiseptics and germicides, denatured alcohol	IMSS	Milliliter	Tub	20,000.00	$679.89	$0.03	Consumable
Lubricating gel	SS	Gram	0	-	$-	$0.04	Consumable
Ambrosol	IMSS	Milliliter	Packet	100.00	$5.20	$0.05	Drug
Swabs	IMSS	Gram	0	500.00	$27.69	$0.06	Consumable
Tongue blade	IMSS	Item	Bag	500.00	$28.80	$0.06	Consumable
Vitamin B complex	IMSS	Tablet	Packet	30.00	$3.20	$0.11	Drug
Hydroxocobalamin	IMSS	Tablet	Packet	30.00	$3.20	$0.11	Drug
Face masks	SS	Item	0	-	$-	$0.13	Consumable
Diphenidol	IMSS	Tablet	Packet	30.00	$3.91	$0.13	Drug
Ferrous sulfate	IMSS	Tablet	Packet	30.00	$4.20	$0.14	Drug
Ranitidine	IMSS	Tablet	Packet	20.00	$3.11	$0.16	Drug
Chlorphenamine	IMSS	Tablet	Packet	20.00	$3.20	$0.16	Drug
Paracetamol	SS	Item	0	10.00	$1.99	$0.20	Drug
Naproxen	IMSS	Tablet	Packet	30.00	$6.05	$0.20	Drug
Diclofenac	IMSS	Capsule	Packet	20.00	$4.20	$0.21	Drug
Surgical soap	SS	Milliliter	0	-	$-	$0.23	Consumable
Cap	0	0	0	-	$-	$0.24	Consumable
Trimethoprim	IMSS	Tablet	Packet	20.00	$4.83	$0.24	Drug
Omeprazole	IMSS	Tablet	Packet	14.00	$3.45	$0.25	Drug
Multiple sample needle 20 GX1”	SS	Item	0	-	$-	$0.26	Consumable
Sterile gauze	SS	Item	0	200.00	$55.09	$0.28	Consumable
Amplification	IMSS	Tablet	Packet	20.00	$7.55	$0.38	Drug
Ciprofloxacin	IMSS	Tablet	Packet	8.00	$3.70	$0.46	Drug
Amoxicillin	IMSS	Tablet	Packet	12.00	$5.80	$0.48	Drug
Phenazopyridine	IMSS	Tablet	Packet	20.00	$10.35	$0.52	Drug
Hyoscine butylbromide	IMSS	Tablet	Packet	10.00	$5.45	$0.55	Drug
Metamizole	SS	Item	0	-	$-	$0.58	Drug
Benzonatate	IMSS	Blister	Packet	20.00	$11.70	$0.59	Drug

Input	Source	Unit measure	Presentation	Quantity per presentation	Overall cost	Unit cost	Type
10 ml syringes	SS	Item	0	-	$-	$0.90	Consumable
3M Tegaderm dressing 4.4 cm x 4.4 cm	IMSS	0	0	50.00	$49.00	$0.98	Consumable
3M Tegaderm CHG dressing	0	0	0	50.00	$49.00	$0.98	Consumable
Recipient for urine samples	0	Item	0	-	$-	$1.00	Consumable
Disposable gloves	IMSS	Item	Box	100.00	$118.50	$1.19	Consumable
Sterile gloves	0	0	0	100.00	$118.50	$1.19	Consumable
Blood sample tube with EDTA K2 powder (anticoagulant) LILA	SS	Item	0	-	$-	$1.42	Consumable
Amikacin	IMSS	Vial	Packet	1.00	$2.19	$2.19	Drug
Oral electrolytes	SS	Item	0	-	$-	$2.19	Drug
Benzylpenicillin	IMSS	Vial	Packet	1.00	$2.32	$2.32	Drug
Hyoscine butylbromide	IMSS	Vial	Packet	3.00	$8.00	$2.67	Drug
Disposable infusion kit	SS	Item	0	-	$-	$2.70	Consumable
Disposable tissues	SS	Item	0	-	$-	$2.71	Consumable
Stationary	0	Kit	0	-	$-	$3.00	Consumable
5 cm bandage	0	0	0	-	$-	$3.65	Consumable
Swan-Ganz 7 F catheter	SS	Item	0	-	$-	$5.47	Consumable
Punzocat 22 g	0	Item	0	-	$-	$5.47	Consumable
Cistoflo urine bag	SS	Item	0	-	$-	$7.38	Consumable
ROJO Serology tube without anticoagulant	SS	Item	0	-	$-	$10.79	Consumable
Diphenhydramine	IMSS	Vial	Packet	1.00	$12.75	$12.75	Drug
Foley probe	SS	Item	0	-	$-	$16.18	Consumable
Urinalysis	DOF	Study	Study	1.00	$61.00	$61.00	Study
Es	DOF	Study	Study	1.00	$61.00	$61.00	Study
Gasometry	SS	Study	Study	1.00	$113.00	$113.00	Study
LFT	DOF	Study	Study	1.00	$61.00	$61.00	Study
Qs	DOF	Study	Study	1.00	$61.00	$61.00	Study
Es (Hosp)	DOF	Study	Study	1.00	$77.00	$77.00	Study
Gasometry (Hosp)	SS	Study	Study	1.00	$113.00	$113.00	Study
LFT (Hosp)	DOF	Study	Study	1.00	$77.00	$77.00	Study
Qs (Hosp)	DOF	Study	Study	1.00	$77.00	$77.00	Study
Hematic biometry	IMSS	Study	Study	-	$-	$88.00	Study
X ray	DOF	Study	Study	1.00	$155.00	$155.00	Study

Continued...

Input	Source	Unit measure	Presentation	Quantity per presentation	Overall cost	Unit cost	Type
Ultrasound	DOF	Study	Study	1.00	$155.00	$155.00	Study
Ultrasound (Hosp)	DOF	Study	Study	1.00	$155.00	$155.00	Study
X ray (Hosp)	DOF	Study	Study	1.00	$266.00	$266.00	Study
Antibodies	SS	Study	Study	-	$-	$409.77	Study
Echocardiogram	DOF	Study	Study	1.00	$482.00	$482.00	Study
Electrocardiogram	DOF	Study	Study	1.00	$482.00	$482.00	Study
Electroencephalogram	DOF	Study	Study	1.00	$482.00	$482.00	Study
Emergency room visit	IMSS	Session	Visit	1.00	$514.00	$514.00	Service
Primary care visit	IMSS	Session	Visit	-	$-	$559.00	Service
Tomography	DOF	Study	Study	1.00	$1,290.00	$1,290.00	Study
Hospitalization	IMSS	Days	0	-	$-	$5,156.00	Service
Hospitalization in ICU	IMSS	Days	Days	-	$-	$30,360.00	Consumable
Hospitalization in ICU	IMSS	Days	Days	1.00	$30,360.00	$30,360.00	Service

2.9 Real Medical Costs SS

Actions	Description of Input	Unit measure	Unit cost	Quantity	Partial cost	Subtotal	Total Action 3	Remarks
3. Treatment Group Outpatients							$424.88	
	**Visit to family doctor (up to 5 visits)**							
	**a) Professional services**					$355.73		
	Primary care visit	Session	$222.98	1.555600	346.8677			
	Emergency room visit	Session	$38.40	0.230700	8.8589			
	**b) Medical consumables**					$5.45		
	Multiple sample needle 20 GX1”	Item	$0.26	1.690000	0.4394			
	Disposable gloves	Item	$0.56	1.770000	0.9912			
	Face masks	Item	$0.17	2.100000	0.3570			
	Sterile gauze	Item	$0.04	2.500000	0.1000			
	Uro-Labstix strip	Item	$1.05	1.110000	1.1655			
	Blood collection tube with EDTA K2 powder (anticoagulant) LILA	Item	$1.42	1.690000	2.3998			
	**c) Drugs and related substances**					$2.72		
	Paracetamol	Item	$0.16	8.580800	1.3729			
	Ibuprofen	Tablet	$0.20	0.160000	0.0320			
	Amoxicillin	Vial	$1.82	0.300000	0.5460			
	Ampicillin	Tablet	$0.08	0.112000	0.0092			
	Ceftriaxone	Vial	$10.19	0.056000	0.5706			
	Benzylpenicillin	Vial	$1.82	0.017500	0.0319			
	Diphenidol	Tablet	$0.12	0.120000	0.0146			
	Doxicyclin	Tablet	$0.09	0.080000	0.0074			
	Erythromycin	Tablet	$0.07	0.144000	0.0100			
	Hyoscine butylbromide	Tablet	$0.14	0.600000	0.0864			
	Methochlopramide	Tablet	$0.02	0.040000	0.0008			
	Tmp/Smx	Tablet	$0.33	0.090000	0.0293			
	Vitamins	Tablet	$0.04	0.036000	0.0015			
	Metamizole	Item	$0.58	0.012000	0.0070			
	Ranitidine	Tablet	$0.05	0.072000	0.0039			

Actions	Description of Input	Unit measure	Unit cost	Quantity	Partial cost	Subtotal	Total Action 3	Remarks
3. Treatment Group Outpatients	**d) Laboratory studies**					$60.98		
	Hematic biometry	Study	$49.31	0.728000	35.8977			
	Gasometry	Study	$113.00	0.036000	4.0680			
	LFT	Study	$105.60	0.056000	5.9136			
	ES	Study	$57.90	0.020000	1.1580			
	QS	Study	$105.60	0.132000	13.9392			
4. Treatment Hospitalization							$6,396.83	
	**Hospitalization**							
	**a) Professional services**					$5,599.00		
	Hospitalization	Days	$1,404.22	3.987265	5,598.9972			
	**b) Medical consumables**					$137.71		
	Tongue blade	Item	$0.10	1.470000	0.1470			
	Blood collection tube with EDTA K2 powder (anticoagulant) LILA	Item	$1.42	1.500000	2.1300			
	Multiple sample needle 20 GX1”	Item	$0.26	1.360000	0.3536			
	Disposable gloves	Item	$0.56	1.920000	1.0752			
	Recipient for urine samples	Item	$1.00	1.080000	1.0800			
	Uro-Labstix strip	Item	$1.05	1.110000	1.1655			
	Foley probe	Item	$8.46	1.000000	8.4600			
	Cistoflo urine bag	Item	$8.46	1.000000	8.4600			
	Lubricating gel	Gram	$0.04	189.900000	7.5960			
	Sterile gauze	Item	$0.04	5.250000	0.2100			
	Surgical soap	Milliliter	$0.23	305.100000	70.1730			
	Face masks	Item	$0.17	2.180000	0.3706			
	Disposable tissues	Item	$3.45	2.000000	6.9000			
	Insulin syringe	Item	$0.61	1.170000	0.7137			
	10 ml syringes	Item	$0.90	1.580000	1.4220			
	Swabs	Gram	$0.05	4.830000	0.2415			
	Heparin	Item	$12.54	1.000000	12.5400			
	Antiseptics and germicides, denatured alcohol	Milliliter	$0.01	204.900000	2.0490			
	ROJO Serology tube without anticoagulant	Item	$10.79	1.170000	12.6243			

Actions	Description of Input	Unit measure	Unit cost	Quantity	Partial cost	Subtotal	Total Action 3	Remarks
4. Treatment Hospitalization	**c) Drugs and related substances**					$21.10		
	Alpha-methyldopa	Tablet	$0.39	0.117978	0.0460			
	Amoxicillin	Vial	$1.82	0.058989	0.1074			
	Hyoscine butylbromide	Vial	$2.33	0.021067	0.0490			
	Cefuroxime	Dose	$18.66	0.033708	0.6290			
	Ciprofloxacin	Dose	$21.78	0.370787	8.0757			
	Doxycycline	Tablet	$0.09	0.884831	0.0823			
	Enterogermina	Vial	$1.00	0.023596	0.0236			
	Phenazopyridine	Tablet	$0.11	0.042135	0.0045			
	Ibuprofen	Tablet	$0.20	0.012472	0.0025			
	Loratadine	Tablet	$0.06	0.019663	0.0011			
	Metoclopramide	Vial	$1.00	0.025281	0.0252			
	Saline solution	Bag	$7.20	1.500000	10.8000			
	Paracetamol	Tablet	$0.16	7.823596	1.2518			
	**d) Laboratory studies**					$639.03		
	LFT	Study	$105.60	0.978000	103.2768			
	Qs	Study	$105.60	1.253000	132.3168			
	Gasometry	Study	$113.00	0.185000	20.9050			
	Es	Study	$57.90	0.820000	47.4780			
	X ray	Study	$268.89	0.216292	58.1588			
	Electroencephalogram	Study	$268.89	0.011236	3.0212			
	Tomography	Study	$268.89	0.002809	0.7553			
	Echocardiogram	Study	$268.89	0.002809	0.7553			
	Electrocardiogram	Study	$268.89	0.117978	31.7230			
	Ultrasound	Study	$270.97	0.348315	94.3828			
	Hematic biometry	Study	$49.31	2.966000	146.2535			

Continued...

Actions	Description of Input	Unit measure	Unit cost	Quantity	Partial cost	Subtotal	Total Action 3	Remarks
5. Treatment in Intensive Care Unit							$69,860.76	
	**Intensive care**							
	**a) Professional services**					$69,465.60		
	Hospitalization in ICU	Days	$8,683.20	8.000000	69,465.6000			
	**b) Medical consumables**					$17.84		
	Tongue blade	Item	$0.10	2.000000	0.2000			
	Multiple sample needle 20 GX1”	Item	$0.26	1.910000	0.4966			
	Swabs	Gram	$0.05	6.640000	0.3320			
	Face masks	Item	$0.17	4.550000	0.7735			
	Antiseptics and germicides, denatured alcohol	Milliliter	$0.01	300.000000	3.0000			
	Disposable gloves	Item	$0.56	4.640000	2.5984			
	Disposable infusion kit	Item	$2.70	1.330000	3.5910			
	Punzocat 22 g	Item	$5.47	1.250000	6.8375			
	Adhesive tape	Centimeter	$0.00	2.180000	0.0065			
	**c) Drugs and related substances**					$18.19		
	Saline solution	Bag	$7.20	2.500000	18.0000			
	Paracetamol	Item	$0.16	1.200000	0.1920			
	**d) Laboratory studies**					$359.13		
	Qs	Study	$105.60	2.000000	211.2000			
	Hematic biometry	Study	$49.31	3.000000	147.9300			

Input	Source	Unit measure	Presentation	Quantity per presentation	Overall cost	Unit cost	Type
Tongue blade	0	Item	Item	1.00	$-	$0.10	Consumable
Multiple sample needle 20 GX1”	SS	Item	0	1.00	$-	$0.26	Consumable
Antiseptics and germicides, denatured alcohol	SS	Milliliter	0		$-	$0.01	Consumable
Swan-Ganz 7 F catheter	SS	Item	0	1.00	$-	$5.47	Consumable
Cistoflo urine bag	SS	Item	0	-	$-	$8.46	Consumable
Face masks	SS	Item	0	1.00	$-	$0.17	Consumable
Disposable infusion kit	SS	Item	0	1.00	$-	$2.70	Consumable
Recipient for urine samples	0	Item	0	1.00	$-	$1.00	Consumable
Sterile gauze	SS	Item	0	1.00	$-	$0.04	Consumable
Lubricating gel	SS	Gram	0	1.00	$-	$0.04	Consumable
Disposable gloves	SS	Item	0	1.00	$-	$0.56	Consumable
Sterile gloves	SS	Item	0	1.00	$-	$0.72	Consumable
Heparin-coated syringe	SS	Item	0	1.00	$-	$12.54	Consumable
Hospitalization in ICU	SS	Days	Days	-	$-	$8,683.20	Consumable
Surgical soap	SS	Milliliter	0	-	$-	$0.23	Consumable
Insulin syringe	IMSS	Item	Item	1.00	$-	$0.61	Consumable
10 ml syringes	SS	Item	Item	1.00	$-	$0.90	Consumable
Disposable tissues	SS	Item	0	-	$-	$3.45	Consumable
Stationary	0	Kit	0	-	$-	$3.00	Consumable
3M Tegaderm dressing 4.4 cm x 4.4 cm	0	0	0	-	$-	$2.00	Consumable
Punzocat 22 g	0	Item	0	-	$-	$5.47	Consumable
Saline solution	0	Milliliter	0	-	$-	$0.02	Consumable
Hartman solution	SS	Milliliter	0	1,000.00	$10.00	$0.01	Consumable
0.9% saline solution	SS	Milliliter	0	-	$-	$0.02	Consumable
Foley probe	SS	Item	0	-	$-	$8.46	Consumable
Adhesive tape	SS	Centimeter	0	-	$-	$0.00	Consumable
Uro-Labstix strip	IMSS	Item	Bottle	100.00	$95.00	$1.05	Consumable
Swabs	SS	Gram	0	-	$-	$0.05	Consumable
Blood sample tube with EDTA K2 powder (anticoagulant) LILA	SS	Item	0	-	$-	$1.42	Consumable
ROJO Serology tube without anticoagulant	SS	Item	0	-	$-	$10.79	Consumable
5 cm bandage	0	0	0	-	$-	$2.00	Consumable
Antibodies	SS	Study	0	-	$-	$409.77	Study

Input	Source	Unit measure	Presentation	Quantity per presentation	Overall cost	Unit cost	Type
Hematic biometry	SS	Study	0	-	$-	$49.31	Study
Echocardiogram	SS	Study	0	1.00	$268.89	$268.89	Study
General urine examination	SS	Study	0	1.00	$70.16	$70.16	Study
Electrocardiogram	SS	Study	0	1.00	$268.89	$268.89	Study
Electroencephalogram	SS	Study	0	1.00	$268.89	$268.89	Study
ES	SS	Study	0	1.00	$57.90	$57.90	Study
Gasometry	SS	Study		1.00	$113.00	$113.00	Study
Liver profile	SS	Study		1.00	$105.60	$105.60	Study
Qs	SS	Study	0	1.00	$105.60	$105.60	Study
X ray	SS	Study	0	1.00	$268.89	$268.89	Study
Tomography	SS	Study	0	1.00	$268.89	$268.89	Study
Ultrasound	SS	Study	0	1.00	$270.97	$270.97	Study
Alpha-methyldopa	IMSS	Tablet	Bottle	30.00	$11.70	$0.39	Drug
Amoxicillin	SS	Vial	Box	1.00	$1.82	$1.82	Drug
Ampicillin	SS	Tablet	Box	20.00	$1.65	$0.08	Drug
Benzylpenicillin	SS	Vial	Box	1.00	$1.82	$1.82	Drug
Hyoscine butylbromide	SS	Vial	Box	3.00	$6.98	$2.33	Drug
Hyoscine butylbromide	SS	Tablet	Packet	10.00	$1.44	$0.14	Drug
Ceftriaxone	SS	Vial	Box	1.00	$10.19	$10.19	Drug
Cefuroxime	SS	Dose	Packet	1.00	$18.66	$18.66	Drug
Ciproflox	SS	Capsule	Bottle	8.00	$5.50	$0.69	Drug
Ciprofloxacin	SS	Dose	Packet	1.00	$21.78	$21.78	Drug
Chlorphenamine	0	0	0	-	$-	#¡DIV/0!	Drug
Vitamin B complex	0	0	0	-	$-	#¡DIV/0!	Drug
Diclofenac	0	0	0	-	$-	#¡DIV/0!	Drug
Diphenidol	SS	Tablet	Packet	30.00	$3.65	$0.12	Drug
Doxicyclin	SS	Tablet	Packet	10.00	$0.93	$0.09	Drug
Doxycycline	SS	Tablet	Packet	10.00	$0.93	$0.09	Drug
Oral electrolytes	SS	Item	0	-	$-	$2.07	Drug
Enterogermina	0	Vial	0	-	$-	$1.00	Drug
Erythromycin	SS	Tablet	Packet	20.00	$1.39	$0.07	Drug
Phenazopyridine	SS	Tablet	Packet	20.00	$2.14	$0.11	Drug
Ibuprofen	SS	Tablet	Bottle	-	$-	$0.20	Drug
Loratadine	SS	Tablet	Packet	20.00	$1.14	$0.06	Drug

Continues...

…Contintued

Input	Source	Unit measure	Presentation	Quantity per presentation	Overall cost	Unit cost	Type
Metamizole	SS	Item	0	-	$-	$0.58	Drug
Metoclopramide	SS	Vial	Packet	6.00	$5.97	$1.00	Drug
Methochlopramide	SS	Tablet	Packet	20.00	$0.40	$0.02	Drug
Naproxen	0	0	0	-	$-	#¡DIV/0!	Drug
Omeprazole	0	0	0	-	$-	#¡DIV/0!	Drug
Paracetamol (acetaminophen)	SS	Item	0	-	$-	$0.16	Drug
Ranitidine	SS	Tablet	Packet	20.00	$1.09	$0.05	Drug
Ferrous sulfate	0	0	0	-	$-	#¡DIV/0!	Drug
Tmp/Smx	SS	Tablet	Packet	20.00	$6.52	$0.33	Drug
Trimethoprim	0	0	0	-	$-	#¡DIV/0!	Drug
Vitamins	SS	Tablet	Packet	30.00	$1.22	$0.04	Drug
Primary care visit	SS	Session	Visit	-	$-	$222.98	Service
Emergency room visit	SS	Session	Visit	1.00	$38.40	$38.40	Service
Hospitalization	SS	Days	0	-	$-	$1,404.22	Service

Continued...
